# Function and Morphology of the Meibomian Glands Using a LipiView Interferometer in Rotating Shift Medical Staff

**DOI:** 10.1155/2020/3275143

**Published:** 2020-08-17

**Authors:** Jing Zhang, Zhengzheng Wu, Liangnan Sun, Xin-hua Liu

**Affiliations:** ^1^Shenzhen Eye Hospital, Shenzhen Key Laboratory of Ophthalmology, Affiliated Shenzhen Eye Hospital of Jinan University, Shenzhen. No. 18, Zetian Road, Futian District, Shenzhen 518040, Guangdong, China; ^2^Department of Ophthalmology, Sichuan Academy of Medical Sciences & Sichuan Provincial People's Hospital, Chengdu 610072, China

## Abstract

**Purpose:**

To investigate the function and morphology of meibomian glands (MG) in night shift medical staff (MS).

**Methods:**

Sixty-two eyes of 31 patients in the MS group and 59 eyes of 31 patients in the control group were consecutively enrolled. All participants completed Ocular Surface Disease Index (OSDI) and Standard Patient Dry Eye Evaluation (SPEED) questionnaires for dry eye severity, as well as Schirmer I and tear break-up time (TBUT) tests. LipiView® II Ocular Surface Interferometer was used for lipid layer thickness (LLT), MG dropout, and partial blink (PB) rate tests. MG expression was measured with an MG evaluator.

**Results:**

The OSDI score in the MS group was 22.39 ± 13.42, which was significantly higher than that in the control group (9.87 ± 6.64 *Z* = −3.997, *P*=0.001). The SPEED score in the MS group was 7.94 ± 3.81, which was significantly higher than in the control group (3.65 ± 2.11, *Z* = −4.766, *P*=0.001). There was no significant difference in Schirmer I test between the MS group and control group (*Z* = −1.346, *P*=0.178). TBUT in MS group was significantly shorter than that in the control group (*Z* = −5.201, *P*=0.001). The mean LLT of the MS group was 55.02 ± 21.17 nm significantly thinner than that of the control group 72.76 ± 21.62 nm (*Z* = −4.482, *P*=0.001). MG loss occurred in 45.16% of affected eyes in the MS group and 16.13% of affected eyes in the control group, and the difference was statistically significant (*χ*^2^ = 14.352, *P*=0.001). MG yielding liquid secretion and MG yielding secretion score were significantly lower in the MS group than in the control group (*Z* = −3.641, *P*=0.001; *Z* = −3.146, *P*=0.001, resp.). There was a negative correlation between mean LLT and SPEED score (Spearman *r* = −0.363, *P*=0.045).

**Conclusions:**

Night shift MS had a higher incidence of MGD compared to day workers.

## 1. Introduction

Dry eye disease (DED) is one of the most common ocular surface diseases [1]. Previous studies have found the prevalence of dry eye to be 6.8% in American adults, 17.9% in Korean elderly, 31.40% in China, and 32% in northern India [2–5]. DED is a multifactorial disease, of which meibomian gland dysfunction (MGD) is one of the main causes [6]. There are several overlapping risk factors between DED and MGD, including female gender, topical medications, contact lens wear, refractive surgery, and demodicosis [7]. Environmental and occupational factors are strongly associated with DED [8]. Occupational conditions of exposure to adverse environments (including driving, smoking, air conditioning/heating, dust, and cooking fumes) account for more than half of dry eye patients in the hospital's underlying population [8].

Shift work is very common in modern society. About 20% of the working population in industrialized countries needs to rotate night work [9]. In particular, rotating night shifts is a requirement for the majority of medical staff (MS). Previous studies have shown that shift work leads to circadian rhythm disturbances and several cardiovascular risk diseases, such as hypertension, high triglyceride levels, and metabolic syndrome [10].

A major problem faced by shift workers is sleep disturbance, including lack of sleep, difficulty falling asleep, and not feeling refreshed after sleeping [11]. Sleep deprivation (SD) is considered as one of the risk factors for DED [5]. Sleep duration of ≥9 h/d has been found to be a protective factor for dry eye symptoms [12]. Patients with obstructive sleep apnea syndrome (OSAS) have a tendency to develop dry eye [13]. In addition, the prevalence of sleep and mood disorders was higher in patients with DED than in patients with other ocular surface diseases, and the severity of sleep quality was correlated with the grade of DED [14, 15]. Improvement in sleep quality has been shown to be beneficial in DED patients [16]. The purpose of this study was to investigate the ocular surface health of MS who regularly work night shifts and evaluate their dry eye tendency.

## 2. Subjects and Methods

### 2.1. Study Design and Patients

Participants were recruited through public advertisements in Sichuan Academy of Medical Sciences and Sichuan Provincial People's Hospital between January 2018 and July 2018. The inclusion criteria for MS group were as follows: the occupation being physicians or nurses, age from 18 to 40 years, with more than half year of night shift work experience (working at nights 12 h for at least three times per month). Inclusion criteria for the control group were as follows: age from 18 to 40 years, daytime workers with regular routines, without night shift rotation, and without sleep disorders. Participants in both groups were excluded for the following reasons: history of ocular trauma; history of ocular surgery; history of regular contact lens wear; active eye diseases (e.g., blepharitis and conjunctivitis); systemic diseases that may affect the ocular surface (e.g., autoimmune diseases, diabetes and thyroid disease, and hyperlipidemia); use of ocular medications within a week, including creams, ointments, or artificial tears; and pregnant or lactating women. All the study procedures were performed in accordance with the principles of the World Medical Association Declaration of Helsinki. Ethical approval was obtained from the Ethics Committee of Sichuan Academy of Medical Sciences and Sichuan Provincial People's Hospital (ethical approval number: 2018210).

### 2.2. Ocular Surface Parameters Assessments

The Ocular Surface Disease Index (OSDI) and the Standard Patient Dry Eye Evaluation (SPEED) questionnaire were used to evaluate eye discomfort symptoms [17–20]. The OSDI assesses the frequency of ocular discomfort symptoms, changes in vision-related quality of life, and environmental triggers during the week prior to the assessment [18]. OSDI scores are graded from 0 to 12 (no symptoms), 13 to 32 (mild and moderate symptoms), and 33 to 100 (severe symptoms) [18]. The SPEED questionnaire was designed to explore the frequency and severity of ocular symptoms and the timing of their appearance [19]. SPEED scores are graded from 0 to 5 (no symptoms), 6 to 14 (mild and moderate symptoms), and 15 to 28 (severe symptoms) [19, 20].

Tear break-up time (TBUT) was measured using fluorescein sodium test strips (Tianjin Jingming New Technology Development Co., Ltd., China). Subjects were asked to look upwards and a drop of a saline-moistened fluorescent strip was placed on the lower palpebral conjunctiva and measured after several blinks. The time interval between the last blink and the first black spot (tear film defect) that appeared after the fluorescein was evenly distributed over the cornea was recorded as TBUT (seconds). Record the average of the 3 measured TBUT. BUT less than 10 seconds indicates tear film instability. Schirmer I test was used to measure the secretory function of the main lacrimal gland. In the absence of topical anesthesia, a standard 5 mm × 35 mm Schirmer strip was placed in the medial and lateral third of the lower fornix with the eye blinking naturally for 5 min. Wet zone length less than 5 mm was considered as inadequate tear secretion.

**Figure 1:**
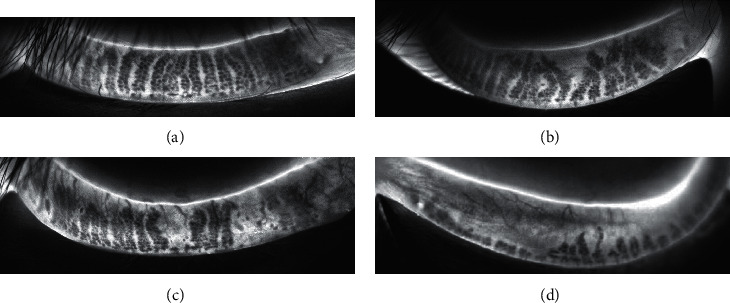
LipiView® II images of penetrating infrared light source for different MG dropout degrees. (a) Grade 0 (without loss of MG). (b) Grade 1 (loss of <33% of the total MG area). (c) Grade 2 (loss of MG area between 33% and 67%). (d) Grade 3 (loss of >67% of the entire MG area).

Lipid layer thickness (LLT), partial blink (PB), and MG morphology were assessed using a LipiView® II Ocular Surface Interferometer (TearScience, Inc. Morrisville, NC, USA). After adjustment to the appropriate sitting position, patients were instructed to fixate on a flashing light source with natural blinking and to acquire the LLT and PB within 20 seconds. Following eversion of the eyelids, lower lid gland imaging was acquired using a LipiView ® II. ImageJ software was used to measure total MG area and MG dropout area. The MG loss rate = MG dropout area/total MG area. MG dropout degree was graded according to the following scale (Figure 1): grade 0 (without loss of MG), grade 1 (loss of <33% of the total MG area), grade 2 (loss of MG area between 33% and 67%), and grade 3 (loss of >67% of the entire MG area).

An MG evaluator (TearScience, Inc.) was applied to observe MG liquid secretions. This was performed with a stable pressure of 3 psi for 10 to 15 seconds at 1-2 mm below the lower eyelid margin. A total of 15 glands were observed in three locations (nasal, middle, and temporal) of the lower eyelid, with five glands in each location [21]. The MG secretion quality of each gland was scored from 0 to 3 (3: clear liquid secretion, 2: colored liquid secretion, 1: concentrated, similar to toothpaste). MG yielding liquid secretion (MGYLS), MG yielding clear liquid secretion (MGYCS), and MG yielding secretion score (MGYSS) were recorded from all 15 glands of the lower eyelid. MGYLS ≦6 or MGYSS ≦18 represents a clear oil secretion dysfunction in MG.

Subjects completed the tests in the following order: first, each participant signed informed consent, completed the OSDI and SPEED questionnaires, and provided general information. Second, a LipiView® II Ocular Surface Interferometer measurement was performed to obtain both PB rate and LLT. Third, TBUT and Schirmer I test were performed, and then MG secretion was measured by MG evaluators. Finally, MG morphology images of the lower eyelid were obtained using a LipiView® II Ocular Surface Interferometer. At least 10 min should elapse between each examination. Patients should rest with eyes closed during the examination interval.

### 2.3. Statistical Analyses

Data analysis was performed using IBM SPSS statistics version 19 statistical software (SPSS Inc., Chicago, IL, USA). BMI was compared between the two groups using the independent-sample *t*-test. The comparison of age, TBUT, Schirmer I, LLT, PB rate, MGYLS, MGYCS, MGYSS, OSDI score, and SPEED score between the two groups was performed using the Wilcoxon signed-rank test. Comparison of gender and MG dropout between the two groups were performed using the Chi-square test. The correlation among the cumulative days of night work, SPEED score, OSDI score, TBUT, Schirmer I value, mean LLT, and MG loss in MS group was analyzed using the Spearman test. All tests were two-tailed, and *P* values less than 0.05 were considered statistically significant.

## 3. Results

### 3.1. Subject Characteristics Comparison

Sixty-two eyes of 31 participants (including 25 physicians and 6 nurses) in the MS group and 59 eyes of 31 participants in the control group were included in this study. The comparison of subject characteristics and various ocular parameters between MS group and the control group is shown in Table 1. Body mass index (BMI) was calculated as weight (kg) divided by height squared (m). There were no significant differences in BMI between MS group and the control group (*t* = −0.517, *P*=0.205). However, the gender difference between the two groups was statistically significant (Chi-square test, *χ*^2^ = 6.458, *P*=0.011). The mean age of the MS group was 26.55 ± 3.15 years (range 20 to 36 years), which was slightly higher than 21.91 ± 4.33 years (range 18 to 33 years) of the control group (*Z* = −4.106, *P*=0.001).

### 3.2. Dry Eye Parameters comparison

The OSDI questionnaire scores for asymptomatic, mild-to-moderate, and severe symptoms in the MS group and the control group were 16.1% versus 54.8%; 54.8% versus 45.2%; and 25.8% versus 0%, respectively. The OSDI score in the MS group was 22.39 ± 13.42, which was significantly higher than 9.87 ± 6.64 in the control group (*Z* = −3.997, *P*=0.001). The SPEED questionnaire scores for asymptomatic (35.5% vs. 83.9%), mild-to-moderate (58.1% vs. 16.1%), and severe (6.5% vs. 0%) symptoms were compared in the MS group and the control group. The SPEED score in the MS group was 7.94 ± 3.81, which was significantly higher than 3.65 ± 2.11 in the control group (*Z* = −4.766, *P*=0.001). TBUT in the MS group was significantly shorter than that in the control group (*Z* = −5.201, *P*=0.001). However, there was no significant difference in Schirmer I test between the two groups (*Z* = −1.346, *P*=0.178).

### 3.3. MGD Parameters comparison

**Figure 2:**
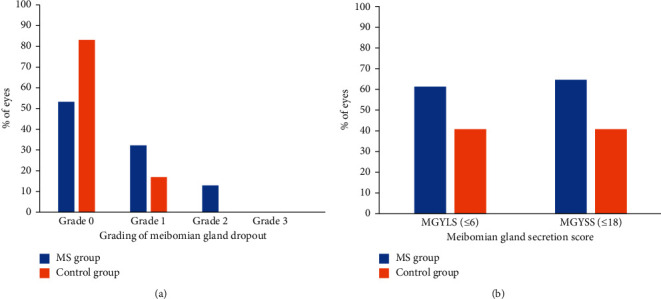
Comparison of meibomian gland dropout grading (a) and meibomian gland secretion score (b) between medical staff (MS) group and control group.

LLT was quantified using mean interference color units (ICU) (1 ICU = 1 nm). Max ICU, mean ICU, and min ICU were significantly thinner in the MS group than in the control group (*Z* = −4.356, *P*=0.001; *Z* = −4.482, *P*=0.001; *Z* = −4.414, *P*=0.001, resp.). Mean LLT ≤60 nm accounted for 69.35% of the eyes in the MS group and 33.87% of the eyes in the control group. MG dropout had occurred in 45.16% of the affected eyes in the MS group and in 16.13% of the affected eyes in the control group. The MG dropout grading for both groups is presented in Figure 2(a). There was significant difference between the two groups for MG dropout (Chi-square test, *χ*^2^ = 14.352, *P*=0.001). There was no statistically significant difference in PB rate between MS group and control group (*Z* = −0.622, *P*=0.534). Both MGYLS and MGYSS were significantly lower in the MS group than in the control group (*Z* = −3.641, *P*=0.001; *Z* = −3.146, *P*=0.002, resp.). However, there was no significant difference in MGYCS between the two groups (*Z* = −1.587, *P*=0.112). MGYLS ≦6 and MGYSS ≦18 for both groups are shown in Figure 2(b).

### 3.4. Correlation of Ocular Surface Parameters

The right eyes of the MS were selected to study the correlations of the ocular surface parameters. The cumulative days of night shift work in the MS group were 142.26 ± 112.07 (36,480) days, which were not correlated with SPEED score, OSDI score, TBUT, Schirmer I value, mean LLT, and MG loss (Spearman *r* = 0.141, 0.274, 0.195, −0.241, 0.042, 0.192, all *P* > 0.05). The mean LLT was negatively correlated with SPEED score (Spearman *r* =  −0.363, *P* > 0.045) but not significantly correlated with Schirmer I, TBUT, or OSDI score and MG loss (Spearman *r* = 0.142, −0.044, −0.346, 0.042, *P*=0.447, 0.815, 0.057, 0.823, all *P* > 0.05).

## 4. Discussion

MGD is the main cause of evaporative DED [22]. Aging is one of the risk factors for MGD [23]. The prevalence of MGD in the Asian population was approximately 33% in patients aged <30 years and 72% in patients aged ≥60 years [4]. In our study, although MS working night shifts were slightly older than day workers, the participants were all young adults aged 18–36 years. We further investigated the association of age with ocular surface parameters and showed no association of age with mean LLT and MG loss. Therefore, we believe that risk factors associated with shift work may exacerbate MGD.

As expected, in this study we found that MS who regularly worked night shifts had more severe and frequent dry eye symptoms than those daytime workers. Although tear secretion function was normal, 90.32% of the affected eyes had shortened TBUTs, 69.35% of the affected eyes had LLT ≤60 nm, and 45% of the affected eyes had MG dropout. LLT measurement was used as a diagnostic tool for MGD [24]. A previous study found LLT ≤60 nm in 74% of patients with severe dry eye symptoms [25]. MS with frequent night shifts causes tear film instability but does not affect tear secretion. Therefore, dry eye in MS is not water deficit but excessive evaporation. Shift work primarily results in sleep disturbances, with acute symptoms including difficulty falling asleep, reduced sleep duration, and somnolence in the following days [11]. Previous studies have reported that sleep disorders and DED interact with each other [13–16]. Poor sleep quality has been reported in 45% of young and middle-aged DED office workers [26]. Fifty patients diagnosed with OSAS were reported to have significantly higher OSDI scores, shorter TBUT, and lower Schirmer values compared to control subjects [13]. SD throughout the day in healthy men caused tear hyperosmolarity, shortened TBUT, and decreased tear secretion, while all SD-induced changes in ocular surface parameters returned to normal levels after one day [12]. Decreased tear secretion and lacrimal gland hypertrophy induced by SD 10-days mice can also be restored to relatively normal levels at 2 weeks [27]. The researches indicate that short-term effects of SD can be compensated. Another study found that dry eye symptoms, tear film instability, and conjunctival hyperemia were aggravated after night shifts in 50 hospital staffs, but the basal Schirmer test increased [28]. They concluded that increased Schirmer test values may be a compensation for stress caused by SD [28]. In our study, the examination of the MS group was not performed on the day of the end of the night shift work, but on other normal working days to avoid temporary effects of SD. Since the tear secretion function of the MS group was not affected in our study, we believe that the secretion function of their lacrimal glands may be compensated or even unaffected.

Previous studies have found an increased incidence of metabolic syndrome such as elevated blood pressure, elevated triglyceride and glucose levels, low HDL cholesterol, and abdominal overweight in shift workers compared to day workers [29, 30]. Shift work was also associated with breast cancer, cardiovascular disease, and pregnancy complications [31–33]. Night shift work disturbs the circadian system, alters sleep activity patterns, and inhibits melatonin production, and these changes can promote inflammation and tumorigenesis and have immunosuppressive effects [31–33]. Inadequate sleep has been shown to be associated with feeling tired and stressed and pessimism [27]. Shift work MS has a high incidence of psychosocial problems such as depression, stress, anxiety, and sleep disorders [34]. Psychosocial problems have been reported to be involved in the neuropathic mechanisms of DED [34].

In our study, the cumulative days of night shift work were not associated with DED parameters and MGD parameters. The SPEED score of night shift MS was negatively correlated with mean LLT, and the more severe and frequent the dry eye condition, the thinner the lipid layer, which was consistent with the previous studies [25]. Different intensity and frequency of night shift work and mental stress of MS in different departments may affect the changes of ocular surface parameters. Dry eye is an inflammatory condition [35]. Cortisol is an important anti-inflammatory hormone in human physiology [36]. Shift work disrupts cortisol production, a marker of HPA axis activation under stress conditions, and may be an important cause of dry eye [37]. Poor sleep quality has been reported to be associated with lower testosterone levels, affecting the development of dry eye [38]. Whether MS with night shift affects different hormone expressions that influence the formation of dry eye still needs further study.

This study has several limitations. First, only single-center MS members were included in this study, which may have selection bias. Second, night shift working hours and assessment of sleep quality were not accurately recorded. Third, most front-line middle-aged MS need to work night shifts, so MS who do not work night shifts were not used as controls in this study. Fourth, the effect of different night shift working modes on the ocular surface needs further analysis.

In conclusion, we should pay much attention to the ocular surface health of rotating shift medical workers. Early treatment of the asymptomatic phase of MGD may delay progression to the symptomatic phase and reverse its pathological progression.

## Figures and Tables

**Table 1:** Comparison between medical staff (MS) group and control group.

	MS group	Control group	*P* value
N/eyes	31/62	31/59	
M : F	10:21	20:11	0.011^&^
Age (y)	26.55 ± 3.15	21.91 ± 4.33	0.001^*∗*^
BMI	20.92 ± 2.00	21.19 ± 2.72	0.607^#^
*DE parameters*			
TBUT(s)	4.63 ± 3.58	7.83 ± 3.33	0.001^*∗*^
Schirmer I (mm)	19.97 ± 10.17	17.77 ± 6.58	0.178^*∗*^
SPEED (score)	7.94 ± 3.81	3.65 ± 2.11	0.000^*∗*^
OSDI (score)	22.39 ± 13.42	9.87 ± 6.64	0.001^*∗*^
*MGD parameters*			
Max ICU (nm)	76.03 ± 18.73	90.06 ± 13.91	0.001^*∗*^
Mean ICU (nm)	55.02 ± 21.17	72.76 ± 21.62	0.001^*∗*^
Min ICU (nm)	44.58 ± 18.42	58.23 ± 19.28	0.001^*∗*^
PB rate (%)	63.72 ± 35.81	68.18 ± 35.29	0.534^*∗*^
MGYLS (score)	5.43 ± 3.98	8.33 ± 4.50	0.001^*∗*^
MGYCS (score)	4.68 ± 4.09	6.00 ± 4.74	0.112^*∗*^
MGYSS (score)	15.48 ± 11.78	22.50 ± 13.01	0.002^*∗*^

MS: medical staff; N: number; M: male; F: female; BMI: body mass index; TBUT: tear film break-up time; SPEED: standard patient evaluation of eye dryness; OSDI: Ocular Surface Disease Index; ICU: interferometry color units; PB: partial blink; MGD: meibomian gland dysfunction; MGYLS: meibomian gland yielding liquid secretion; MGYCS: meibomian gland yielding clear liquid secretion; and MGYSS: meibomian gland yielding secretion score. ^&^Chi-square test; ^*∗*^Wilcoxon signed-rank test; ^#^Independent-sample *t*-test.

## Data Availability

All data used to support the findings of this study are available from the corresponding author upon request.
